# Transcriptional Responses of *Treponema denticola* to Other Oral Bacterial Species

**DOI:** 10.1371/journal.pone.0088361

**Published:** 2014-02-05

**Authors:** Juni Sarkar, Ian H. McHardy, Emil J. Simanian, Wenyuan Shi, Renate Lux

**Affiliations:** 1 School of Dentistry, University of California, Los Angeles, Los Angeles, California, United States of America; 2 Department of Pathology and Laboratory Medicine, University of California, Los Angeles, Los Angeles, California, United States of America; 3 Department of Microbiology, Immunology, and Molecular Genetics, University of California, Los Angeles, Los Angeles, California, United States of America; University of Cape Town, South Africa

## Abstract

The classic organization by Socransky and coworkers categorized the oral bacteria of the subgingival plaque into different complexes. *Treponema denticola*, *Porphyromonas gingivalis and Tannerella forsythia* are grouped into the red complex that is highly correlated with periodontal disease. Socransky's work closely associates red with orange complex species such as *Fusobacterium nucleatum* and *Prevotella intermedia* but not with members of the other complexes. While the relationship between species contained by these complexes is in part supported by their ability to physically attach to each other, the physiological consequences of these interactions and associations are less clear. In this study, we employed *T. denticola* as a model organism to analyze contact-dependent responses to interactions with species belonging to the same complex (*P. gingivalis and T. forsythia*), the closely associated orange complex (using *F. nucleatum* and *P. intermedia* as representatives) and the unconnected yellow complex (using *Streptococcus sanguinis* and *S. gordonii* as representatives). RNA was extracted from *T. denticola* alone as well as after pairwise co-incubation for 5 hrs with representatives of the different complexes, and the respective gene expression profiles were determined using microarrays. Numerous genes related to motility, metabolism, transport, outer membrane and hypothetical proteins were differentially regulated in *T. denticola* in the presence of the tested partner species. Further analysis revealed a significant overlap in the affected genes and we identified a general response to the presence of other species, those specific to two of the three complexes as well as individual complexes. Most interestingly, many predicted major antigens (e.g. flagella, Msp, CTLP) were suppressed in responses that included red complex species indicating that the presence of the most closely associated species induces immune-evasive strategies. In summary, the data presented here provide an in-depth understanding of the transcriptional responses triggered by contact-dependent interactions between microorganisms inhabiting the periodontal pocket.

## Introduction

*T. denticola* is considered to be a significant contributor to periodontal disease and its abundance is highly correlated with periodontal pocket depth, an important indicator of disease severity [1], [2], [3]. While numerous potential virulence factors have been identified and reviewed [4], their roles during infection, especially in a multispecies context remain to be elucidated. Similarly advances have been made in understanding signaling events in *T. denticola* triggered by environmental conditions associated with periodontal disease [5], [6], [7], however, the molecular mechanisms associated with its response to other oral bacterial species are largely unknown. This anaerobic spirochete is a member of the “red complex”, which is comprised of *T. denticola*, *T. forsythia*, as well as *P. gingivalis* [8], [9], [10]. Red complex organisms were found to be highly correlated with periodontal lesions [10], thrive in close contact with each other and exhibit synergistic relationships [11], [12]. *T. denticola* does not attach to early colonizing *Streptococci* (yellow complex) and therefore requires interaction with bridging organisms such as *F. nucleatum* and *P. intermedia* (orange complex) for integration into the oral biofilm community [13], [14].

Co-localization and physical association likely facilitate physiologically and biochemically relevant activities between bacteria. Indeed, numerous examples of metabolic interactions have been documented. For example, metabolic cooperation has been observed between *T. denticola* and *P. gingivalis* and both organisms benefit from the presence of the other [15], [16], [17]. However, information about downstream transcriptional regulation in *T. denticola* in response to interactions with other subgingivial bacteria is currently still lacking. While flowcell-based model systems are available for some of the interspecies interactions tested here [18], [19], [20], [21], we chose a simpler coincubation model in which equal numbers of cells are pelleted together. This approach allows testing of all interspecies interactions under similar conditions independent of their ability to form biofilms together *in vitro*, which was relevant especially for the assessment of transcriptional responses towards yellow complex species. In summary, by employing microarray technology, this study is aimed at investigating the transcriptional responses of *T. denticola* during early contact-induced dual species interactions with representatives of different oral complexes.

## Materials and Methods

### Bacterial strains and growth conditions

Treponema denticola ATCC 35405, Fusobacterium nucleatum ATCC 23726, Streptococcus sanguinis ATCC 10556, Streptococcus gordonii ATCC 10558, Porphyromonas gingivalis W83 and Prevotella intermedia ATCC 49046 were cultivated in TYGVS medium [22], while T. forsythia ATCC 43037 was grown in new oral spirochete (NOS) medium supplemented with vitamin K (0.2 µg/ml) and N-acetylmuramic acid (0.01 µg/ml) [23]. Cells were grown in either 15 ml or 50 ml centrifuge tubes in an anaerobic chamber (5% CO_2_, 5% H_2_ and 90% N_2_) at 37°C. For these experiments, cell numbers were selected to ensure that sufficient quantities of mRNA was obtainable, and thus ∼5×10^9^ cells of each organism were used in all dual-species co-incubations. Further, conditions were first selected to ensure physical contact between selected organisms, and thus organisms were mixed at a 1∶1 ratio. Co-incubation experiments were performed as follows: 5×10^9^ cells, as enumerated with a Petroff-Hausser bacterial counting chamber, of each bacterial species were pelleted at 4,600×g for 10 mins at room temperature and then resuspended in 5 ml of pre-reduced TYGVS. Bacteria were combined such that T. denticola was paired with each of the other species listed above at a 1∶1 ratio in 10 ml of pre-reduced TYGVS. Dual species suspensions were then pelleted again at 4,600×g for 10 minutes at room temperature, placed into the anaerobic chamber, and incubated as pellets at 37°C for 5 hrs to capture transcriptional changes during the early stages of interaction. “Unpaired” T. denticola was treated identically as a control. All experiments were performed in triplicate.

### RNA extraction and purification

After 5 hrs of incubation, supernatants were removed from pelleted bacteria. RNA was extracted using Trizol® Plus Reagent (Invitrogen, CA, USA) according to the manufacturer's instructions. Extracted RNA was treated with DNAse I (Ambion, NY, USA) to remove residual genomic DNA. RNA samples were then further purified using the RNeasy Mini kit (Qiagen, CA, USA) according to the manufacturer's protocol. cDNA was analyzed using *T. denticola* species specific 16 s rRNA gene primers to confirm absence of genomic DNA contamination from the isolation process. qPCR with species-specific 16 s rRNA gene primers was employed to assess the level of RNA from the different interacting partner species isolated along with *T. denticola* RNA. This extraction procedure was found to be differentially selective for extraction from *T. denticola* as compared to other species, which, when extracted individually using similar numbers of cells, resulted in <50% of the total RNA extracted from *T. denticola*.

### Fluorescent cDNA preparation

For all microarray experiments, 5 µg of control or experimental RNA was combined with 5 µg of random hexamers and hybridized at 70°C for 10 mins. Reverse transcription was performed using Superscript III (Invitrogen, NY, USA) as described previously [6]. RNA was hydrolyzed in the presence of 0.1 M EDTA and 0.2 N NaOH at 65°C for 10 mins. A final concentration of 0.3 M HEPES pH 7.5 was added to buffer the reactions. cDNA was further purified and concentrated using Microcon-30 filters (Millipore. MA, USA) and sodium bicarbonate (pH 9.0) was added to a final concentration of 0.1 M. Three μl of 1 mM Amersham mono-reactive Cy™3 and Cy™5 (GE Healthcare, CA, USA) dyes were incubated with the corresponding cDNA samples in the dark for 1 hr at room temperature. Labeled cDNA was then purified with Wizard® SV Gel and PCR Clean-Up System (Promega, WI, USA) according to the manufacturer's protocol.

### Microarray hybridization and analysis

Microarrays were obtained through the NIAID's Pathogen Functional Genomics Resource Center, managed and funded by the Division of Microbiology and Infectious Diseases, NIAID, NIH, DHHS and operated by the J. Craig Venter Institute. Each microarray experiment was performed in triplicate with control cDNA labeled with Cy3 and test cDNA labeled with Cy5. One array for each condition was used in a dye-swapping experiment to address the possible effects of labeling bias. Freshly purified labeled test and control cDNA were combined prior to incubation with hybridization solution (1×: 3× SSC, 24 mM HEPES (pH 7.0), 0.225% SDS) at 95°C for 2 mins. Samples were then evenly dispersed onto microarray slides with cover-slips by capillary action and placed into hybridization chambers. Hybridization chambers were sealed and incubated at 48°C for 12 hrs. Labeled arrays were washed twice sequentially with the following 3 solutions for 10 mins each: Solution 1 (low stringency) contained 2× SSC and 0.1% SDS and was heated to 55°C prior to washing the slides. Solution 2 (medium stringency) contained 0.1× SSC and 0.1% SDS. Solution 3 (high stringency) contained 0.1× SSC. Slides were briefly washed with water, dried and scanned with a Genepix 4000A scanner (MDS, CA, USA).

Fluorescence intensities of each spot were calculated using Genepix Pro, version 6.0 (MDS, CA, USA). The program's morphological opening background subtraction was used to reduce noise and each array was normalized such that the average normalized ratio of medians was equal to one. The four in-slide replicates from each slide were combined. The resulting 12 replicates for each gene were normalized such that the average normalized ratio of medians of each spot in the combined list was equal to one. The data sets were subjected to statistical analysis using Significance Analysis of Microarray (SAM) software under an academic license from Stanford University [24]. Delta values were chosen such that the false discovery rates were ∼5%. Induced and repressed genes were extrapolated from significance lists generated by SAM by identifying the average ratio of median value of the replicates for each gene and selecting genes that had log values above 2 or below -2. Fold regulation shown in all tables is the average ratio of median value for each gene. The ORFs adjacent to the genes meeting above cut-off criteria were further analyzed in the context of possible operons based on the annotations available in the KEGG (www.kegg.jp) and Oralgen (www.oralgen.lanl.gov/) databases. Genes predicted to be organized in the same operons as the genes identified using the original cut-off that exhibited the same trend of differential expression in the presence of the partner species tested were then included in our dataset. Expression patterns of these differentially regulated operons were then compared regarding their response in the presence of each partner species tested and considered for analysis if the gene exhibited induction/repression with log values above 1.5 or below -1.5. Data presented are in compliance with MIAME requirements. Microarray data were deposited on MIAMExpress (http://www.ebi.ac.uk/miamexpress/) with the accession number: E-MEXP-3059.

To assess potential cross reactivity with non-treponemal cDNA, cDNA was generated for all other test organisms used in this study and subjected individually to the same hybridization procedure described above. cDNA from these organisms, excluding *T. denticola*, produced very low background levels of hybridization, indicating little to no cross reactivity was occurring.

### Real-time quantitative PCR

Twelve genes were selected that represented various levels of microarray-predicted induction or repression for all tested conditions. PCR primers (Table 1) that specifically amplified products of 90–120 bp in length for each gene were designed using PrimerQuest (IDT, CA, USA). RNA was reverse transcribed using the transcriptor first strand cDNA synthesis kit (Roche, Basel, Switzerland) and the resulting cDNA was diluted 1∶50 for each PCR reaction. Quantitative PCR was performed with a MyiQ Real Time PCR Detection System (Biorad, CA, USA) and the accompanying program Biorad iQ5 using SYBR Green (Biorad, CA, USA) according to the manufacturer's protocol. Before analysis, RT-qPCR data was normalized across all samples using the abundance of cDNA produced by the 16 s rRNA of *T. denticola* as quantified with species-specific primers. Comparison between microarray and qPCR generated data for the same genes resulted in a fit of correlation based on R^2^ value <0.3 (Figure S1).

**Table 1:** Primers used in this study.

Gene ID	Forward primer 5′-3′	Reverse primer 5′-3′
TDE0358	GGAGCATGGCATTGCTGCATACAT	AACAAATCCGCCTTGGCTTTCTCC
TDE0405	AGATTTGGTCACCTATCCGCGACA	AGGTCATCGCTTGCATAACCGAGT
TDE0449	TTGGATGCAGGAGCAAGCTAAGGA	TTCCGTATTCGGTACTTTGGGCAC
TDE1004	TTTACGTATTAACCGAGCGGGCGA	TGCTTGGTTCAAACCGCGAATCTG
TDE1028	AGTGACAGCTTAAAGAGCCGACTCAC	TACTAAAGCACCTCCTGCTTATAAGTTAC
TDE1029	TACGGACAGCGTATTTGATGCCCT	GCATTCCGCAGCTTGCATTCTTGA
TDE1072	GATGATGAACTTGCAATGGGCGGT	GCAAAGGCAAAGGCATACCTGACA
TDE1238	AAGCAATTCGGCCTTCGGCTCAAA	CAGTCGGTTGACGTTTCGGTTTGT
TDE1408	TGGGCTTATCAGGCTGTTGGAAGT	TGGTGGGAACAACATCTACCCAGT
TDE1548	TGTATCGGGCGGAGGTCTTGTAAA	TGAGCAGCCCTGACTAAATCCTGA
TDE1722	CAAGGAGAGGTAACCATCCAGTTA	TCTCCGGCTTCTGCTGTAATTCT
TDE2009	GCTAAGCGCATAAGCGGTTCATCA	GTTTATAATCGTCCACCTTGCGGC
16 s rRNA	TAATACCGAATGTGCTCATTTACAT	CTGCCATATCTCTATGTCATTGCTCTT

## Results

A comprehensive microarray analysis of interactions between *T. denticola* and representative members of selected oral complexes is summarized below. Initial analyses revealed that the extent and nature of *T. denticola* responses varied depending on the partner species tested (Table 2). Notably, differential gene regulation triggered by either *P. gingivalis* or *T. forsythia* of the red complex largely overlapped, while there was little similarity in response pattern between the two species each tested for the yellow and orange complexes. These sets of differentially regulated genes were then further examined to identify global as well as complex- and species-specific responses in *T. denticola* to the presence of the partner species tested.

**Table 2:** Extend of differential gene regulation in *T. denticola* in response to partner strains.

Complex	Yellow		Orange		Red	
Partner strain	*Sg*	*Ss*	*Fn*	*Pi*	*Pg*	*Tf*
Total *	59 (41)	8 (8)	17 (14)	81 (43)	94 (61)	109 (79)
Induced *	24 (22)	8 (8)	7 (7)	59 (30)	7 (7)	5 (5)
Repressed *	35 (19)	0 (0)	10 (7)	22 (13)	87 (54)	104 (74)
Overlap within complex *	3 (3)		6 (4)		83 (56)	

* genes (operons). The following abbreviations were used for the species: Yellow complex: *Sg - S. gordonii; Ss - S. sanguinis*; Orange complex: *Fn - F. nucleatum; Pi - P. intermedia*; Red complex *Pg - P. gingivalis; Tf - T. forsythia*.

### General response to the presence of bacterial species from the red, orange or yellow complexes

**Figure 1:**
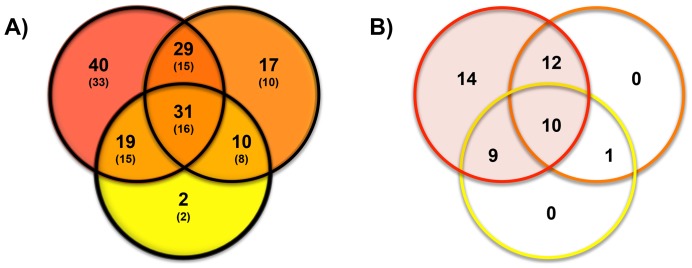
Venn Diagram of A) transcriptional responses of *T. denticola* to the presence of the red complex members *P. gingivalis* and *T. forsythia* (total of 119 genes in 79 operons), the orange complex members *F. nucleatum* and *P. gingivalis* (total of 87 genes in 49 operons) and the yellow complex members *S. gordonii* and *S. sanguinis* (total of 62 genes in 41 operons); B) distribution of genes with predicted antigenic properties among the transcriptional response of *T. denticola* presented in A). Differentially expressed genes that overlap with genes predicted to have antigenic properties according to Veith *et. al* 2009 are highlighted with an asterisk in Table S1. Complexes are indicated by red, orange and yellow color of the circles for A) and the lines for B).

First, we examined the obtained microarray data sets for a general response to the presence of other species regardless if they were members of the red complex (*P. gingivalis* and *T. forsythia*), the closely associated orange complex (*F. nucleatum* and *P. intermedia*) or the more distant yellow complex (*S. gordonii* and *S. sanguinis*) [10]. Of the 148 total *T. denticola* genes that were found to respond to above partner species (Table S1), 31 genes (16 operons) were differentially regulated by at least one representative of each complex and thus considered to be part of a general response to the presence of other bacteria (Table 3 and Figure 1A). Only ten of these genes were repressed, while the remaining 21 exhibited either repression or induction depending on the partner species. The predicted and known functions of the generally repressed genes included cell surface features such as the major outer sheath protein (TDE0405) and several flagella-related proteins (and TDE1408/09 to TDE1474/75), as well as the glycine cleavage pathway (TDE1624-27) and a hypothetical protein (TDE0718). In contrast to the other flagella-associated genes, the flagellar filament core protein encoding ORF TDE1004 was induced by the presence of the orange complex member *P. intermedia* and repressed in the presence of the other species it responded to. Expression of *msp* and two of the *fla* genes (TDE1004 and TDE1408) was further validated by RT-qPCR (Table S2). Among the genes with mixed responses, the hypothetical proteins encoding ORFs (TDE0059, TDE0226, TDE1155, and TDE2214) as well as TDE2300 (PDZ domain protein) were differentially regulated in a pattern that showed repression by the red complex species and induction by members of the other complexes. Several ribosomal and translation-related functions including two associated conserved hypothetical proteins (TDE0766-69, TDE0790-93, TDE0881-85, and TDE1677/78) also responded to the presence of most representatives of the different oral complexes tested in this study (Table 3).

**Table 3:** Overlapping response in *T. denticola* to members of the tested oral complexes.

Complex			Yellow		Orange		Red	
Locus	Gene Symbol	Predicted Gene Product	*Sg*	*Ss*	*Fn*	*Pi*	*Pg*	*Tf*
TDE0059		hypothetical protein	**2.42**			1.63		−1.69
TDE0226		hypothetical protein		1.66	1.63			−**2.20**
TDE0405		major outer sheath protein	−**2.92**		−1.52	−1.66		−**2.12**
TDE0718		hypothetical protein	−1.60		−1.89	−**2.25**	−**2.10**	−**2.26**
TDE0766	*rpsJ*	ribosomal protein S10	-			**2.02**	−**2.32**	−1.78
TDE0767	*rplC*	ribosomal protein L3	−1.57	1.52		+	−**2.20**	−**2.69**
TDE0768	*rplD*	ribosomal protein L4	-	+		1.84	−**2.41**	−**2.22**
TDE0769		ribosomal protein L23	−1.60	+		+	−**2.13**	−1.95
TDE0790		ribosomal protein S11	**2.54**			-	+	+
TDE0791	*rpoA*	DNA-directed RNA polymerase, alpha subunit	-			**2.40**	−1.73	−1.80
TDE0792	*rplQ*	ribosomal protein L17	-			**2.20**	-	−1.58
TDE0793		conserved hypothetical protein	-			**2.22**	-	-
TDE0881	*rpsP*	ribosomal protein S16	−1.83			1.90	-	−**2.26**
TDE0882		conserved hypothetical protein	−1.72			**2.04**	-	-
TDE0883		16S rRNA processing protein RimM	−1.61			1.51	−1.53	−1.62
TDE0884		tRNA (guanine-N1)-methyltransferase	−1.66			-	-	-
TDE0885		ribosomal protein L19	-			1.67	−1.65	−1.66
TDE1004		flagellar filament core protein	−**2.23**			1.58	−1.54	−**2.02**
TDE1155		hypothetical protein	**2.48**			1.74		−**2.00**
TDE1408		flagellar filament outer layer protein FlaA, putative	−1.66		−1.62		−**2.38**	−**3.45**
TDE1409		flagellar filament outer layer protein FlaA, putative	−1.73		−1.67		−1.96	−1.91
TDE1474		hypothetical protein	−1.82		-		−1.67	−**2.10**
TDE1475		flagellar filament core protein	−1.56		−1.63		-	−**2.50**
TDE1624	*gcvP2*	glycine cleavage system P protein, subunit 2	−**2.05**		−1.68	−**2.23**	−**2.31**	−**2.03**
TDE1625	*gcvP1*	glycine cleavage system P protein, subunit 1	−1.82		-	−**2.17**	−**2.32**	−**2.08**
TDE1626	*gcvH*	glycine cleavage system H protein	−1.77		-	−1.63	−**2.23**	−**2.17**
TDE1627	*gcvT*	glycine cleavage system T protein	-		−1.59	−1.81	−**2.30**	−**2.46**
TDE1677	*ssb*	single-strand binding protein	−1.52	1.50		**2.06**	−**2.27**	−**2.34**
TDE1678	*rpsF*	ribosomal protein S6				**2.10**	−**2.32**	−**2.22**
TDE2214		conserved hypothetical protein		1.63		1.67	−1.88	−2.68
TDE2300		trypsin domain/PDZ domain protein	1.96			**2.42**	−1.55	−1.67

For species abbreviations see Table 2. Numbers highlighted in bold indicate the original cutoff of log 2/−2 for regulated genes. Numbers that are not in bold represent genes that are predicted to be organized in an operon with at least one gene meeting the log 2/−2 cutoff in the presence of at least one of the partner species tested with a regulation of log between 1.5/−1.5 and log 2/−2. Differential regulations that did not meet the cutoff criteria within an operon are included as + (to indicate induction) or – (to indicate repression) to reflect if they followed the overall trend of gene regulation in an operon.

### Overlapping transcriptional responses of *T. denticola* to two of the three oral complexes tested

#### Overlap between the red complex and orange complex

*T. denticola* is a member of the red complex which is closely associated with the orange complex [10]. To investigate if this association is reflected in the transcriptional response of *T. denticola* to the presence of these species, we analyzed the expression data for overlapping responses to representatives of these two complexes (Table 4) that were not already part of the general response analyzed above (Table 3). With 29 affected genes, the size of this response category was similar to the general response described above. Among these one gene was induced and 11 genes were repressed by at least one representative of each the orange and red complexes, while the remaining 17 were induced by the orange complex member they responded to and repressed by the red complex as detailed below. Only one operon consisting of three hypothetical/conserved domain encoding proteins (TDE2465-67) exhibited a response to all four of the individually tested partner species. The majority of differentially regulated genes responded to the presence of *P. intermedia* and the red complex species. Closer examination of the expression pattern revealed that ORFs TDE0761/62 (dentilisin protease complex), TDE1072 (lipoprotein), TDE1978-80 (hypothetical genes), TDE2200 (methionine lyase) were repressed by all three species. In contrast, ORFs TDE1238 (preprotein translocase), TDE1272-74 (part of a larger ABC transporter gene operon spanning from TDE1271-75), TDE1482, TDE2054-56 (Hemin binding protein encoding genes), TDE2078-80 (regulatory genes), and TDE2601/02 (outer membrane proteins) were all induced by *P. intermedia* but repressed by red complex species. The ferritin encoding ORF TDE0449 was the only gene induced by the presence of *P. intermedia* as well as *T. forsythia*. On the other hand, TDE0295 (*gyrA*), TDE1477 (flagellar filament core) and TDE2180 (*tmrE*) were affected by *F. nucleatum* as well as either *P. gingivalis* or *T. forsythia*.

**Table 4:** Overlapping response in *T. denticola* to members of the Orange and Red complexes.

Complex			Orange		Red	
Locus	Gene Symbol	Predicted Gene Product	*Fn*	*Pi*	*Pg*	*Tf*
TDE0295	*gyrA*	DNA gyrase, A subunit	**5.46**			−1.53
TDE0449		ferritin, putative		1.52		**5.62**
TDE0761	*prcA*	protease complex-associated polypeptide		-	−**2.21**	−1.72
TDE0762	*prcB*	serine protease, dentilisin, authentic frameshift		−1.55	−1.79	−1.71
TDE1072		lipoprotein, putative		−**2.72**	−**2.67**	−**2.64**
TDE1238	*secG*	preprotein translocase, SecG subunit		1.83	−**2.38**	−**3.05**
TDE1271		oligopeptide/dipeptide ABC transporter, ATP-binding protein		1.56		
TDE1272		oligopeptide/dipeptide ABC transporter, ATP-binding protein		**2.00**	−1.80	−1.62
TDE1273		oligopeptide/dipeptide ABC transporter, peptide-binding protein		**2.25**	−1.89	-
TDE1274		oligopeptide/dipeptide ABC transporter, permease protein		**2.10**	−**2.18**	−**2.12**
TDE1275		oligopeptide/dipeptide ABC transporter, permease protein		1.57		
TDE1477		flagellar filament core protein	−1.75		−1.77	−**2.23**
TDE1482		peptidase, M24 family protein		1.75	−**2.00**	−**2.16**
TDE1978		conserved hypothetical protein		−**2.12**	-	-
TDE1979		hypothetical protein		−**2.14**	−1.53	−1.58
TDE1980		hypthetical protein		−1.57	+	-
TDE2054		conserved hypothetical protein		**2.68**	−1.86	−1.89
TDE2055	*hbpB*	hemin-binding protein B		**2.43**	−**2.11**	−**2.00**
TDE2056		outer membrane hemin-binding protein A		**2.98**	−**2.29**	−**2.85**
TDE2078		TPR domain protein		1.53	-	−1.58
TDE2079		sigma-54 dependent transcriptional regulator, putative		1.68	-	-
TDE2080		cytidylate kinase/ribosomal protein S1		**2.10**	−1.98	−**2.05**
TDE2180	*trmE*	tRNA modification GTPase TrmE	1.52		−1.50	−**3.04**
TDE2200	*megL*	methionine gamma-lyase		−**2.78**	−**2.24**	−1.86
TDE2465		hypothetical protein	-	−**2.90**	−1.61	−1.77
TDE2466		conserved hypothetical protein	−1.53	−**3.30**	−1.58	−1.85
TDE2467		conserved domain protein	−1.67	−**2.59**	−1.87	−1.91
TDE2601		surface antigen, putative		1.52	-	-
TDE2602		outer membrane protein, putative		1.59	−1.71	−**2.01**

See legend Table 3.

#### Overlap between the red complex and yellow complex

Even though the species organized in the red and yellow complexes have been classified as not being closely associated [10], a considerable overlap in differential gene expression between the presence of yellow and red complex species was observed (Table 5). Interestingly, the majority of the 19 genes that overlap between the responses to the different species tested were regulated in the presence of either one or both of the red complex species as well as *S. gordonii*. Most of the 15 affected genes following this pattern spanned a variety of cellular functions encoded by ORFs TDE011 (peroxiredoxin), TDE0842-44 (cytoplasmic filament protein, hypothetical protein and pyruvate phosphate kinase), TDE0855 (response regulator), TDE1171 (hypothetical protein), TDE2119/20 (glycine reductase proteins), and TDE2508 (hypothetical protein) and were repressed during co-incubation with *S. gordonii* or either one/both members of the red complex representatives. ORFs TDE1830 (hypothetical protein), TDE1961 (PIN domain protein) and TDE2429 (hypothetical protein) were induced by the presence of these species, while TDE0358 (*cinI*) and TDE1722 (hypothetical protein) were induced by *S. gordonii* but repressed by *T. forsythia*. Similarly, TDE0237 (HDIG domain protein), TDE1663/64 (OmpA and a conserved domain protein) as well as TDE2369 (conserved domain protein) were induced by the presence of *S. sanguinis* and repressed by both of the red complex species tested in this study.

**Table 5:** Overlapping response in *T. denticola* to members of the Red and Yellow complexes.

			Yellow		Red	
Locus	Gene Symbol	Predicted Gene Product	*Sg*	*Ss*	*Pg*	*Tf*
TDE0011		alkyl hydroperoxide reductase/peroxiredoxin	−1.82		−**2.01**	−**2.34**
TDE0237		HDIG domain protein		1.51	−1.85	−**2.28**
TDE0358	*cinI*	cinnamoyl ester hydrolase	**4.04**			−**2.12**
TDE0842	*cfpA*	cytoplasmic filament protein A	-		−1.94	−**3.12**
TDE0843		conserved hypothetical protein	−1.81		−1.50	−1.50
TDE0844		pyruvate phosphate dikinase, putative	−1.72		−**2.30**	−**2.21**
TDE0855		DNA-binding response regulator	−1.60		−**2.03**	−**2.14**
TDE1171		conserved hypothetical protein	−1.51		−1.95	−**2.30**
TDE1663		OmpA family protein		1.95	−**2.14**	−**2.04**
TDE1664		conserved domain protein		+	−1.69	−1.93
TDE1722		hypothetical protein	3.30			−1.50
TDE1830		hypothetical protein	1.58			**2.44**
TDE1838		conserved hypothetical protein	2.01		-	-
TDE1961		PIN domain protein	1.77		**4.92**	
TDE2119	*grdB-2*	glycine reductase complex selenoprotein GrdB2	−1.73		−**2.32**	-
TDE2120		glycine reductase complex proprotein GrdE2	−1.65		−**2.29**	−1.93
TDE2369		conserved domain protein		1.52	−**2.11**	−**2.37**
TDE2429		hypothetical protein	2.00		**9.96**	1.67
TDE2508		hypothetical protein	−1.94		−1.84	−**2.19**

See legend Table 3.

#### Overlap between the orange complex and yellow complex

The transcriptional responses of *T. denticola* to representatives of the yellow and orange complexes affected only ten genes (Table 6). The most overlap was observed between *S. gordonii* (yellow complex) and *P. intermedia* (orange complex) but no particular pattern was apparent. The induction of several stress response related genes (*grpE, dnaK, clpB* and *hsp20*) was noticeable.

**Table 6:** Overlapping response in *T. denticola* to members of the Yellow and Orange complexes.

Complex			Yellow		Orange	
Locus	Gene Symbol	Predicted Gene Product	*Sg*	*Ss*	*Fn*	*Pi*
TDE0082		transcriptional regulator, MerR family	1.99			−**2.28**
TDE0197		PIN domain protein	**2.46**			−**2.17**
TDE0627		co-chaperone protein GrpE	1.68	+		**2.33**
TDE0628	*dnaK*	chaperone protein DnaK	1.91	1.59		**2.40**
TDE0904		hypothetical protein	1.64		**2.84**	
TDE1028		hypothetical protein	+			**4.96**
TDE1029		Hsp20/alpha crystallin family protein	**2.34**			-
TDE1226	*troA*	zinc ABC transporter, periplasmic zinc-binding protein	**2.29**			**4.04**
TDE1556		conserved domain protein	**2.00**			1.88
TDE2327	*clpB*	ATP-dependent Clp protease, ATP-binding subunit ClpB	**2.20**			1.53

See legend Table 3.

### Specific responses to individual complexes

#### Red complex species

The most extensive transcriptional response in *T. denticola* to individual complexes was observed in the presence of other representatives of the red complex (Table 7). Interestingly, the majority of the affected genes were repressed, while induction was only observed for *cobM* (TDE0614) in response to *P. ginigivalis* or *T. forsythia* as well as for TDE1516 (ABC transporter) and TDE2118 (topoisomerase IV) in the presence of *P. ginigivalis*. Genes repressed in response to either one or both members of the red complex include ORFs encoding cellular processes (TDE0076, TDE0110, TDE0200, TDE0665, TDE2001, TDE2235/36, TDE2271, TDE2326 and TDE2739), membrane-associated functions (TDE0586, TDE1246, TDE1386, TDE1712, TDE1947, TDE1950, TDE2217 and TDE2232-34) as well as hypothetical proteins (TDE0111/12, TDE0753/54, TDE1231, TDE1460, TDE1717, TDE2285, TDE2315, TDE2557 and TDE2674).

**Table 7:** Response in *T. denticola* to members of the Red complex.

Complex			Red	
Locus	Gene Symbol	Predicted Gene Product	*Pg*	*Tf*
TDE0076		aldolase, DeoC/FbaB family	−1.82	−2.12
TDE0110		M23/M37 peptidase domain protein	−1.89	−**2.29**
TDE0111		conserved hypothetical protein	−1.52	−1.81
TDE0112		conserved hypothetical protein	-	-
TDE0200		tetrapyrrole methylase family protein	−1.72	−**2.35**
TDE0308		hypothetical protein		−**2.56**
TDE0567		hypothetical protein	−1.75	−**2.06**
TDE0586		membrane protein, putative	−1.89	−**2.09**
TDE0614	*cobM*	precorrin-4 C11-methyltransferase	**2.91**	**2.83**
TDE0665		pyruvate ferredoxin/flavodoxin oxidoreductase family protein	−**2.24**	−**2.22**
TDE0693	*thiD*	phosphomethylpyrimidine kinase	**3.25**	
TDE0745	*grdA*	glycine reductase complex selenoprotein GrdA		−**2.37**
TDE0753		hypothetical protein	−**2.03**	−1.84
TDE0754		hypothetical protein	−**2.03**	−1.78
TDE1231		hypothetical protein	−1.55	−**2.13**
TDE1246		lipoprotein, putative	−1.54	−1.57
TDE1247		hypothetical protein	−1.54	−**2.28**
TDE1386		methyl-accepting chemotaxis protein	-	−**2.39**
TDE1460		conserved domain protein		−**2.40**
TDE1516		ABC transporter, ATP-binding protein, putative	**2.23**	
TDE1712	*flaA*	flagellar filament outer layer protein		−**2.05**
TDE1717		hypothetical protein	−1.93	−**2.88**
TDE1947		ABC transporter, permease protein	-	−**2.18**
TDE1950	*tmpC*	membrane lipoprotein TmpC, putative	−**2.43**	−1.66
TDE2001		oligoendopeptidase F, putative		−**2.31**
TDE2118		topoisomerase IV, A subunit, putative	**3.72**	
TDE2217	*mglB*	galactose/glucose-binding lipoprotein	−**2.09**	−**2.20**
TDE2217	*mglB*	galactose/glucose-binding lipoprotein	−**2.09**	−**2.20**
TDE2232		iron compound ABC transporter, ATP-binding protein, putative		
TDE2233		iron compound ABC transporter, permease protein, putative		-
TDE2234		iron compound ABC transporter, periplasmic iron compound-binding protein, putative	−1.80	−**2.03**
TDE2235		methylaspartate ammonia-lyase	−**2.38**	−**2.20**
TDE2236		methylaspartate mutase, E subunit	−1.97	−1.82
TDE2271		HAM1 protein		−**2.15**
TDE2285		conserved hypothetical protein	−**2.35**	−**3.14**
TDE2315		conserved hypothetical protein TIGR00044	−1.55	−**2.83**
TDE2326		cobyric acid synthase CobQ, putative		−**2.12**
TDE2557		hypothetical protein		−**2.19**
TDE2674		hypothetical protein	−1.84	−**2.42**
TDE2712		Hypothetical protein	-	−**2.00**
TDE2739		membrane protein, putative	-	−**2.00**

See legend Table 3.

#### Orange complex species

In contrast to the specific responses to the red complex members, there was no overlap observed in the transcriptional changes triggered by the two representatives of the orange complex species tested in this study (Table 8). Only TDE0040 (AMP binding protein) and TDE1548 (conserved hypothetical protein) were specifically induced by *F. nucleatum*. The response observed for TDE1548 was confirmed by RT-qPCR (Table S2). The specific response to *P. intermedia* included induction of several ORFs encoding cellular functions (TDE0163, TDE2399/2400), membrane associated functions (TDE2006-08), and hypothetical proteins (TDE0164, TDE2009 and TDE2398) as well as repression of some cellular processes (TDE0431, TDE1593/94 and TDE2410) and one hypothetical protein TDE2093.

**Table 8:** Response in *T. denticola* members of the Orange complex.

Complex			Orange	
Locus	Gene Symbol	Predicted Gene Product	*Fn*	*Pi*
TDE0040		AMP-binding protein	**2.15**	
TDE0163		Flavodoxin		**3.34**
TDE0164		conserved hypothetical protein		1.73
TDE0431		LysM domain protein		−**2.08**
TDE1548		conserved hypothetical protein TIGR00103	**9.24**	
TDE1593		Fe-hydrogenase		−1.70
TDE1594		pyridine nucleotide-disulphide oxidoreductase family protein		−**2.16**
TDE2006		membrane protein, putative		**2.56**
TDE2007		ABC transporter, ATP-binding/permease protein		**3.17**
TDE2008		ABC transporter, ATP-binding/permease protein		**2.45**
TDE2009		conserved hypothetical protein		**4.03**
TDE2093		conserved hypothetical protein		−**2.30**
TDE2372		conserved hypothetical protein		**2.83**
TDE2398		conserved hypothetical protein		1.92
TDE2399	*rnpA*	ribonuclease P protein component		**2.36**
TDE2400		ribosomal protein L34		1.50
TDE2410		Hemolysin		−**2.29**

See legend Table 3.

#### Yellow complex species

While there was considerable overlap between the transcriptional changes of *T. denticola* in the presence of yellow complex species and red complex species, only TDE0120 (conserved hypothetical protein) and TDE1142 (putative phage minor structural protein) were specifically induced by the yellow complex species *S. gordonii* (Table S1).

## Discussion

In this study, we report for the first time a comprehensive analysis of gene expression profiles in *T. denticola* triggered by the contact with other relevant oral bacterial species including two representatives each for the red (*P. gingivalis* and *T. forsythia*), orange (*F. nucleatum* and *P. intermedia*), and yellow (*S. gordonii* and *S. sanguinis*) complexes. The extent of observed transcriptional responses in *T. denticola* appears to reflect previously established disease-related associations, interspecies interactions and synergistic relationships [10], [13], [25] (Figure 1A). Of the total differentially regulated genes (148 genes in 99 operons) identified in this study, the majority (119 genes in 79 operons) was included in responses to the presence of the other red complex species, *P. gingivalis* or *T. forsythia* (Figure 1A). Most of these responses were specific to the red complex alone (Table 7) followed by the overlapping general response that includes red, orange and complex representatives (Table 3) and the overlapping response triggered by the red as well as orange complexes (Table 4). Common responses towards red and yellow complex species were less prevalent (Table 5). Consistent with above observation that the extent of differential gene expression appears to emulate the association between *T. denticola* and the tested partner species, far fewer transcriptional responses were specifically triggered by the orange complex species (Tables 4, 6 and Figure 1A) and only two were unique to the yellow complex (Figure 1A). Additionally, under the conditions tested with each member of the orange and yellow complexes, it was observed that the response uniquely overlapped between yellow and orange were limited to predicted stress responses (Table 6). Most of these functions were identified in our previous study on responses of *T. denticola* to changes in environmental conditions as being induced by oxygen, osmotic stress, heat and blood [6].

The close association of *T. denticola* with *P. gingivalis* and *T. forsythia* is also reflected by the large overlap in transcriptional responses towards these two red complex partner species tested (Table 2). This finding is consistent with previous studies that have investigated individual virulence factors expressed in *T. denticola* [4], [26] as well as the synergistic interactions between *T. denticola* and either *P. gingivalis* or *T. forsythia* [9], [27], [28]. These species also have been shown to co-aggregate [29], form synergistic biofilms [30], exhibit cooperative proteinase activity [31] and induce IL-6 production in murine macrophages [32]. In contrast, gene expression triggered by the tested partner species from other complexes appeared to be more individualized with little overlap within the complex. However, while most of the differentially regulated genes responded to the presence of red complex species (119 of 148 total), the majority of affected genes were shared with both (31 genes) or either one of the orange (29 genes) and yellow (19 genes) complexes (Figure 1A) albeit not necessarily following the same pattern of induction/repression (Tables 3, 4, 5).

### Regulation of predicted antigens

Another noteworthy observation is the finding that well over 90% of the 119 *T. denticola* genes that were differentially regulated by either one of the red complex species were repressed (Table 2), while the other species tested triggered more balanced responses (*S. gordonii* and *F. nucleatum*) or a bias towards induction (*S. sanguinis* and *P. intermedia*). Most interestingly, comparison of the *T. denticola* genes that responded to the presence of other species with the list of proteins predicted in a study by Veith and coworkers [33] to contain antigenic properties (Tables 3, 4, 5, 7 and Table S1) revealed that almost a third of the 148 differentially genes detected in our study encode possible antigens. Among these 46 antigen-encoding ORFs, 45 were part of the response to the red complex species alone or in overlap with other complexes (Figure 1B). All these genes were repressed with the exception of the ferritin encoding TDE0449 indicating that in the presence of its most closely associated red complex partner species immune evasive strategies are enhanced in *T. denticola*. The presence of the other species tested had a more differential effect and eleven of the predicted antigens were induced. This finding suggests that expression of certain cellular function was more important than antigen suppression for *T. denticola* when in combination with orange or yellow complex members. The chaperone encoding *dnaK* was the only exception among the predicted antigens that was not differentially expressed in response to red complex species, while its induction was triggered by the presence of orange as well as yellow complex members.

#### Virulence factors

Many of the predicted antigens repressed in this study have been previously characterized as being important virulence factors [4], [9], [26]. Examples include the flagellar proteins that were predominantly part of the general response (Table 3). In addition to their antigenic properties [33], [34], reduction in flagella production and thus motility has been proposed to be important for biofilm architecture [35], [36]. The gene *msp* that encodes another well-characterized principal antigen and virulence factor was also repressed by the presence of members of all complexes (Table 3). While reduction of this major antigen facilitates immune evasion, involvement of Msp attachment to other species (in particular *P. gingivalis* and *F. nucleatum*) has been discussed [29], [37] but was found in other studies not to be essential [4], [29], [38], [39]. Under the experimental conditions of our study Msp would have been present to initiate the contact in the beginning of the coincubation period if necessary. After interaction with the respective partner strain is established, reduction of antigenic properties could become the next important cellular response and thus result in *msp* repression at the 5 hr time point that was measured in this study. Msp has been associated with another principal antigen, the potent surface-expressed protease CTLP complex (also known as dentilisin) that exerts cytotoxic effects on host epithelial cells [40], [41]. While *msp* was repressed in *T. denticola* in response to each partner strain tested, the ORFs encoding the antigenic CTLP [42] were repressed only in the presence of members of the orange and red complexes (Tables 3 and 4). Similar to the repression of *msp*, the down-regulation of this important virulence factor suggests that immune evasion may become a priority when closely associated bacterial species are present and the initial contact has been established.

#### Other membrane associated and metabolic proteins

Among the down-regulated genes that overlap with those predicted to have antigenic properties are a number of membrane associated and metabolic proteins that have not been classified as virulence factors for *T. denticola*. These include the antigenic cytoplasmic filament encoding *cfpA*, which is required for establishing a mixed biofilm with *P. gingivalis* [30] and was repressed in the presence of red complex species as well as *S. gordonii* (Table 5). Similar as discussed above for Msp, CfpA function may not be required after contact is established and thus is repressed when immune evasion becomes the more important feature. Proteins involved in glycine metabolism are also among those identified to have antigenic properties due to their membrane-associated components [33]. Glycine degradation is an important metabolic pathway for *T. denticola* [43] and the reductive cleavage of glycine is coupled to ATP synthesis [44], [45], [46]. The apparent importance of reducing surface antigens in the presence of relevant subgingival community partners is underscored by the finding that genes encoding these important function are repressed either as part of the general response like the glycine cleavage pathway (TDE1624-27) (Table 3) or in the presence of red or yellow complex species such as the glycine reductase pathway (TDE2119/20) (Table 5). In addition to induction of an immune evasive response in the presence of other partner species, repression of these proteins would be consistent with a synergistic relationship, which reduces the need for these functions when *T. denticola* is co-incubated with these partner strains. Other membrane-associated or metabolic proteins that follow the same pattern of regulation like *cfpA* or the glycine reductase pathway are the antigenic OmpA (TDE1663), and the peroxiredoxin encoding TDE0011. One of the predicted roles of peroxiredoxin is the defense against oxidative toxins like oxides and peroxides under stress conditions. This was confirmed in our previous study in *T. denticola* that showed significant upregulation of TDE0011 in response to oxygen and osmotic stress as well as blood [6]. In addition to potential antigen reduction, repression of this gene suggests that the presence of red complex species or *S. gordonii* can reduce oxidative stress and is consistent with a synergistic relationship between these species [47]. Suppression of other prominent antigens of *T. denticola* such as MglB (TDE2217) and TmpC (TDE1950) [33], [48], [49] is mediated only upon co-incubation with other red complex species (Table 7).

#### Iron uptake

The expression pattern observed for ORFs encoding iron uptake systems appears to be governed by competition rather than antigen suppression. The ferritin encoding ORF TDE0449 is induced by *T. forsythia* (red complex) as well as *P. intermedia* (orange complex), while the ORFs encoding HbpA and HbpB (TDE2055/56) are repressed in the presence of red complex species but induced after coincubation with *P. intermedia* (Table 4). ORFs TDE2232-36, which encode an iron compound ABC transporter only responded to red complex species and were repressed (Table 7). A differential response in which low affinity iron uptake systems were replaced by high affinity systems to increase competitiveness in the presence of other species was previously described for *P. gingivalis* [49].

In conclusion, our study showed transcriptional regulation of numerous proteins with potential antigenic properties, supporting a synergistic interaction of these oral pathogens in the onset of periodontal infection. Notably, the extent of specific responses of *T. denticola* to bacterial species belonging to different complexes appeared to correlate with their previously described association. From an ecological perspective this could be a reflection of the level of co-evolution between interacting species in a periodontal polymicrobial context.

## Supporting Information

Figure S1 **Correlation between microarray and RT-qPCR generated gene expression values.** Differential expression values for 12 genes were compared when *T. denticola* was in the presence of other species. Trend line shows the best-fit linear regression and the corresponding R^2^ value is indicated.
(TIF)

Table S1 **See legend** **Table 3** **.** * indicates that these genes were identified as putative surface antigens by Veith *et. al* 2009.
(DOCX)

Table S2 **See legend** **Table 3** **.** MA  =  values derived from microarray experiments, RT  =  values derived from real-time PCR experiments
(DOCX)
