# Supplementary figures and images for: Transcriptional Responses of Treponema denticola to Other Oral Bacterial Species
Source: PLoS One. 2014 Feb 5;9(2):e88361. doi: 10.1371/journal.pone.0088361 (PMC3914990; doi:10.1371/journal.pone.0088361)

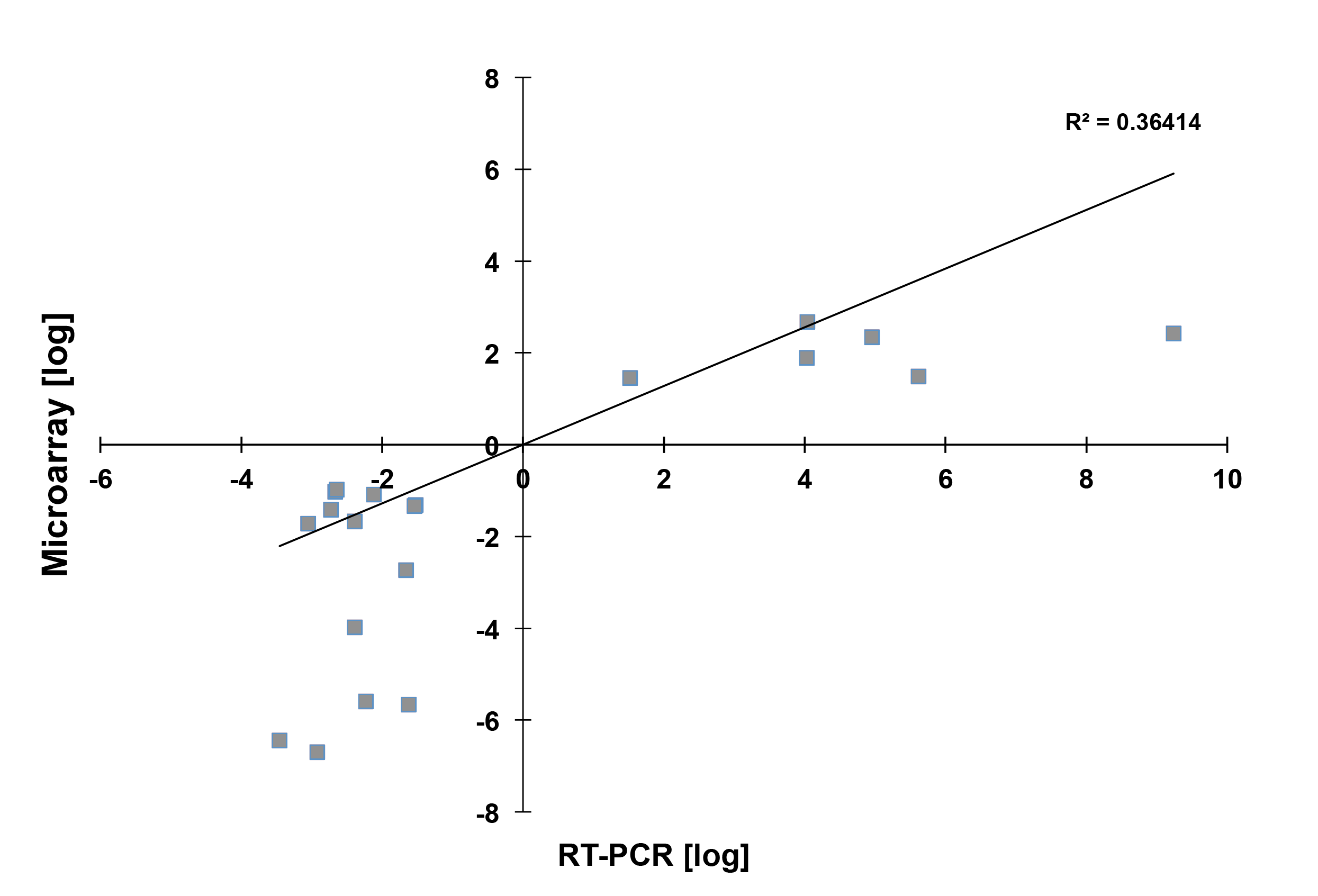

Supplement: Figure S1 — Correlation between microarray and RT-qPCR generated gene expression values. Differential expression values for 12 genes were compared when T. denticola was in the presence of other species. Trend line shows the best-fit linear regression and the corresponding R2 value is indicated. (TIF) [file pone.0088361.s001.tif]
