# Supplementary material for: Transcriptional Responses of Treponema denticola to Other Oral Bacterial Species
Source: PLoS One. 2014 Feb 5;9(2):e88361. doi: 10.1371/journal.pone.0088361 (PMC3914990; doi:10.1371/journal.pone.0088361)
Supplement: Table S1 — See legend Table 3 . * indicates that these genes were identified as putative surface antigens by Veith et. al 2009. (DOCX) [file pone.0088361.s002.docx]

Supplemental Table 1. Transcriptional response in *T. denticola* to members of all oral complexes tested in this study.

|  |  |  | | **Yellow** | | **Orange** | | **Red** | |  |
| --- | --- | --- | --- | --- | --- | --- | --- | --- | --- | --- |
| **Table** | **Locus** | **Symbol** | **Predicted Gene Product** | ***Sg*** | ***Ss*** | ***Fn*** | ***Pi*** | ***Pg*** | ***Tf*** |  |
| 5 | TDE0011* |  | alkyl hydroperoxide reductase/peroxiredoxin | -1.82 |  |  |  | **-2.01** | **-2.34** |  |
|  |  |  |  |  |  |  |  |  |  |  |
| 8 | TDE0040 |  | AMP-binding protein |  |  | **2.15** |  |  |  |  |
|  |  |  |  |  |  |  |  |  |  |  |
| 3 | TDE0059 |  | hypothetical protein | **2.42** |  |  | 1.63 |  | -1.69 |  |
|  |  |  |  |  |  |  |  |  |  |  |
| 7 | TDE0076 |  | aldolase, DeoC/FbaB family |  |  |  |  | -1.82 | **-2.12** |  |
|  |  |  |  |  |  |  |  |  |  |  |
| 6 | TDE0082 |  | transcriptional regulator, MerR family | 1.99 |  |  | **-2.28** |  |  |  |
|  |  |  |  |  |  |  |  |  |  |  |
| 7 | TDE0110 |  | M23/M37 peptidase domain protein |  |  |  |  | -1.89 | **-2.29** |  |
| 7 | TDE0111 |  | conserved hypothetical protein |  |  |  |  | -1.52 | -1.81 |  |
| 7 | TDE0112 |  | conserved hypothetical protein |  |  |  |  | - | - |  |
|  |  |  |  |  |  |  |  |  |  |  |
| 4c | TDE0120 |  | conserved hypothetical protein | **2.73** |  |  |  |  |  |  |
|  |  |  |  |  |  |  |  |  |  |  |
| 8 | TDE0163 |  | Flavodoxin |  |  |  | **3.34** |  |  |  |
| 8 | TDE0164 |  | conserved hypothetical protein |  |  |  | 1.73 |  |  |  |
|  |  |  |  |  |  |  |  |  |  |  |
| 6 | TDE0197 |  | PIN domain protein | **2.46** |  |  | **-2.17** |  |  |  |
|  |  |  |  |  |  |  |  |  |  |  |
| 7 | TDE0200 |  | tetrapyrrole methylase family protein |  |  |  |  | -1.72 | **-2.35** |  |
|  |  |  |  |  |  |  |  |  |  |  |
| 3 | TDE0226 |  | hypothetical protein |  | 1.66 | 1.63 |  |  | **-2.20** |  |
|  |  |  |  |  |  |  |  |  |  |  |
| 5 | TDE0237 |  | HDIG domain protein |  | 1.51 |  |  | -1.85 | **-2.28** |  |
|  |  |  |  |  |  |  |  |  |  |  |
| 4 | TDE0295 | *gyrA* | DNA gyrase, A subunit |  |  | **5.46** |  |  | -1.53 |  |
|  |  |  |  |  |  |  |  |  |  |  |
| 7 | TDE0308 |  | hypothetical protein |  |  |  |  |  | **-2.56** |  |
|  |  |  |  |  |  |  |  |  |  |  |
| 5 | TDE0358 | *cinI* | cinnamoyl ester hydrolase | **4.04** |  |  |  |  | **-2.12** |  |
|  |  |  |  |  |  |  |  |  |  |  |
| 3 | TDE0405* |  | major outer sheath protein | **-2.92** | - | -1.52 | -1.66 | - | **-2.12** |  |
|  |  |  |  |  |  |  |  |  |  |  |
| 8 | TDE0431 |  | LysM domain protein |  |  |  | **-2.08** |  |  |  |
|  |  |  |  |  |  |  |  |  |  |  |
| 4 | TDE0449* |  | ferritin, putative |  |  |  | 1.52 |  | **5.62** |  |
|  |  |  |  |  |  |  |  |  |  |  |
| 7 | TDE0567 |  | hypothetical protein |  |  |  |  | -1.75 | **-2.06** |  |
|  |  |  |  |  |  |  |  |  |  |  |
| 7 | TDE0586 |  | membrane protein, putative |  |  |  |  | -1.89 | **-2.09** |  |
|  |  |  |  |  |  |  |  |  |  |  |
| 7 | TDE0614 | *cobM* | precorrin-4 C11-methyltransferase |  |  |  |  | **2.91** | **2.83** |  |
|  |  |  |  |  |  |  |  |  |  |  |
| 6 | TDE0627 |  | co-chaperone protein GrpE | 1.68 | + |  | **2.33** |  |  |  |
| 6 | TDE0628* | *dnaK* | chaperone protein DnaK | 1.91 | 1.59 |  | **2.40** |  |  |  |
|  |  |  |  |  |  |  |  |  |  |  |
| 7 | TDE0665* |  | pyruvate ferredoxin/flavodoxin oxidoreductase family protein |  |  |  |  | **-2.24** | **-2.22** |  |
|  |  |  |  |  |  |  |  |  |  |  |
| 7 | TDE0693 | *thiD* | phosphomethylpyrimidine kinase |  |  |  |  | **3.25** |  |  |
|  |  |  |  |  |  |  |  |  |  |  |
| 3 | TDE0718 |  | hypothetical protein | -1.60 |  | -1.89 | **-2.25** | **-2.10** | **-2.26** |  |
|  |  |  |  |  |  |  |  |  |  |  |
| 7 | TDE0745 | *grdA* | glycine reductase complex selenoprotein GrdA |  |  |  |  |  | **-2.37** |  |
|  |  |  |  |  |  |  |  |  |  |  |
| 7 | TDE0753 |  | hypothetical protein |  |  |  |  | **-2.03** | -1.84 |  |
| 7 | TDE0754* |  | hypothetical protein |  |  |  |  | **-2.03** | -1.78 |  |
|  |  |  |  |  |  |  |  |  |  |  |
| 4 | TDE0761* | *prcA* | protease complex-associated polypeptide |  |  |  |  | **-2.21** | -1.72 |  |
| 4 | TDE0762* |  | serine protease, dentilisin, authentic frameshift |  |  |  | -1.55 | -1.79 | -1.71 |  |
|  |  |  |  |  |  |  |  |  |  |  |
| 3 | TDE0766 | *rpsJ* | ribosomal protein S10 | - |  |  | **2.02** | **-2.32** | -1.78 |  |
|  |  |  |  |  |  |  |  |  |  |  |
| 3 | TDE0767 | *rplC* | ribosomal protein L3 | -1.57 | 1.52 |  | + | **-2.20** | **-2.69** |  |
| 3 | TDE0768 | *rplD* | ribosomal protein L4 | - | + |  | 1.84 | **-2.41** | **-2.22** |  |
| 3 | TDE0769 |  | ribosomal protein L23 | -1.60 | + |  | + | **-2.13** | -1.95 |  |
|  |  |  |  |  |  |  |  |  |  |  |
|  | TDE0770 | *rplB* | ribosomal protein L2 | - |  |  | 1.71 | - | **-2.18** |  |
|  | TDE0771 |  | ribosomal protein S19 | - |  |  | + | - | - |  |
|  | TDE0772 |  | ribosomal protein L22 | -1.51 |  |  | + | - | - |  |
|  |  |  |  |  |  |  |  |  |  |  |
|  | TDE0773 |  | ribosomal protein S3 | -1.60 |  |  | + | - | - |  |
|  | TDE0774 |  | ribosomal protein L16 | + |  |  | 1.63 | -1.62 | - |  |
|  | TDE0775 |  | ribosomal protein L29 | + |  |  | 1.99 | -1.74 | - |  |
|  | TDE0776 |  | ribosomal protein S17 | -1.52 |  |  | + | -1.53 | - |  |
|  | TDE0777 |  | ribosomal protein L14 | -1.53 |  |  | + | -1.75 | -1.95 |  |
|  | TDE0778 |  | ribosomal protein L24 | - |  |  | 1.78 | -1.75 | -1.69 |  |
|  | TDE0779 |  | ribosomal protein L5 | 1.78 |  |  | - | + | + |  |
|  | TDE0780 | *rpsN* | ribosomal protein S14 | **2.03** |  |  | 1.94 | -1.87 | - |  |
|  | TDE0781 |  | ribosomal protein S8 | -1.77 |  |  | + | + | - |  |
|  | TDE0782 | *rplF* | ribosomal protein L6 | - |  |  | 1.87 | -1.76 | 3.68 |  |
|  | TDE0783 |  | ribosomal protein L18 | -1.61 |  |  | + | + | - |  |
|  | TDE0784 | *rpsE* | ribosomal protein S5 | -1.55 |  |  | + | -1.84 | -2.05 |  |
|  | TDE0785 |  | ribosomal protein L30 | - |  |  | 1.77 | - | -1.53 |  |
|  | TDE0786 |  | ribosomal protein L15 | - |  |  | 1.79 | - | - |  |
|  | TDE0787 |  | preprotein translocase, SecY subunit | - |  |  | 1.56 | -1.62 | - |  |
|  |  |  |  |  |  |  |  |  |  |  |
|  | TDE0788 |  | ribosomal protein L36 | - |  |  | 2.37 | -1.92 | -1.61 |  |
|  | TDE0789 |  | ribosomal protein S13 | -1.56 |  |  | 1.99 | -1.65 | - |  |
|  |  |  |  |  |  |  |  |  |  |  |
| 3 | TDE0790 |  | ribosomal protein S11 | **2.54** |  |  | - | + | + |  |
| 3 | TDE0791 | *rpoA* | DNA-directed RNA polymerase, alpha subunit | - |  |  | **2.40** | -1.73 | -1.80 |  |
| 3 | TDE0792 | *rplQ* | ribosomal protein L17 | - |  |  | **2.20** | - | -1.58 |  |
| 3 | TDE0793 |  | conserved hypothetical protein | - |  |  | **2.22** | - | - |  |
|  |  |  |  |  |  |  |  |  |  |  |
| 5 | TDE0842* | *cfpA* | cytoplasmic filament protein A | - |  |  |  | -1.94 | **-3.12** |  |
| 5 | TDE0843 |  | conserved hypothetical protein | -1.81 |  |  |  | -1.50 | -1.50 |  |
| 5 | TDE0844 |  | pyruvate phosphate dikinase, putative | -1.72 |  |  |  | **-2.30** | **-2.21** |  |
|  |  |  |  |  |  |  |  |  |  |  |
| 5 | TDE0855* |  | DNA-binding response regulator | -1.60 |  |  |  | **-2.03** | **-2.14** |  |
|  |  |  |  |  |  |  |  |  |  |  |
| 3 | TDE0881 | *rpsP* | ribosomal protein S16 | -1.83 |  |  | 1.9 | - | **-2.26** |  |
| 3 | TDE0882 |  | conserved hypothetical protein | -1.72 |  |  | **2.04** | - | - |  |
| 3 | TDE0883 |  | 16S rRNA processing protein RimM | -1.61 |  |  | 1.51 | -1.53 | -1.62 |  |
| 3 | TDE0884 |  | tRNA (guanine-N1)-methyltransferase | -1.66 |  |  | - | - | - |  |
| 3 | TDE0885 |  | ribosomal protein L19 | - |  |  | 1.67 | -1.65 | -1.66 |  |
|  |  |  |  |  |  |  |  |  |  |  |
| 6 | TDE0904 |  | hypothetical protein | 1.64 |  | **2.84** |  |  |  |  |
|  |  |  |  |  |  |  |  |  |  |  |
| 3 | TDE1004* |  | flagellar filament core protein | **-2.23** |  |  | 1.58 | -1.54 | **-2.02** |  |
|  |  |  |  |  |  |  |  |  |  |  |
| 6 | TDE1028 |  | hypothetical protein |  |  |  | **4.96** |  |  |  |
| 6 | TDE1029 |  | Hsp20/alpha crystallin family protein | **2.34** |  |  |  |  |  |  |
|  |  |  |  |  |  |  |  |  |  |  |
| 4 | TDE1072* |  | lipoprotein, putative |  |  |  | **-2.72** | **-2.67** | **-2.64** |  |
|  |  |  |  |  |  |  |  |  |  |  |
| 4c | TDE1142 |  | phage minor structural protein, putative | **2.01** |  |  |  |  |  |  |
|  |  |  |  |  |  |  |  |  |  |  |
| 3 | TDE1155 |  | hypothetical protein | **2.48** |  |  | 1.74 |  | **-2.00** |  |
|  |  |  |  |  |  |  |  |  |  |  |
| 5 | TDE1171 |  | conserved hypothetical protein | -1.51 |  |  |  | -1.95 | **-2.30** |  |
|  |  |  |  |  |  |  |  |  |  |  |
| 6 | TDE1226 | *troA* | zinc ABC transporter, periplasmic zinc-binding protein | **2.29** |  |  | 4.04 |  |  |  |
|  |  |  |  |  |  |  |  |  |  |  |
| 7 | TDE1231* |  | hypothetical protein |  |  |  |  | -1.55 | **-2.13** |  |
|  |  |  |  |  |  |  |  |  |  |  |
| 4 | TDE1238 | *secG* | preprotein translocase, SecG subunit |  |  |  | 1.83 | **-2.38** | **-3.05** |  |
|  |  |  |  |  |  |  |  |  |  |  |
| 7 | TDE1246* |  | lipoprotein, putative |  |  |  |  | -1.54 | -1.57 |  |
| 7 | TDE1247* |  | hypothetical protein |  |  |  |  | -1.54 | **-2.28** |  |
|  |  |  |  |  |  |  |  |  |  |  |
| 4 | TDE1271 |  | oligopeptide/dipeptide ABC transporter, ATP-binding protein |  |  |  | 1.56 |  |  |  |
| 4 | TDE1272 |  | oligopeptide/dipeptide ABC transporter, ATP-binding protein |  |  |  | **2.00** | -1.80 | -1.62 |  |
| 4 | TDE1273* |  | oligopeptide/dipeptide ABC transporter, peptide-binding protein |  |  |  | **2.25** | -1.89 | - |  |
| 4 | TDE1274 |  | oligopeptide/dipeptide ABC transporter, permease protein |  |  |  | **2.10** | **-2.18** | **-2.12** |  |
| 4 | TDE1275 |  | oligopeptide/dipeptide ABC transporter, permease protein |  |  |  | 1.57 |  |  |  |
|  |  |  |  |  |  |  |  |  |  |  |
| 7 | TDE1386 |  | methyl-accepting chemotaxis protein |  |  |  |  | - | **-2.39** |  |
|  |  |  |  |  |  |  |  |  |  |  |
| 3 | TDE1408* |  | flagellar filament outer layer protein FlaA, putative | -1.66 |  | -1.62 |  | **-2.38** | **-3.45** |  |
| 3 | TDE1409* |  | flagellar filament outer layer protein FlaA, putative | -1.73 |  | -1.67 |  | -1.96 | -1.91 |  |
|  |  |  |  |  |  |  |  |  |  |  |
| 7 | TDE1460 |  | conserved domain protein |  |  |  |  |  | **-2.40** |  |
|  |  |  |  |  |  |  |  |  |  |  |
| 3 | TDE1474 |  | hypothetical protein | -1.82 |  | - |  | -1.67 | **-2.10** |  |
| 3 | TDE1475* |  | flagellar filament core protein | -1.56 |  | -1.63 |  | -1.44 | **-2.50** |  |
|  |  |  |  |  |  |  |  |  |  |  |
| 4 | TDE1477* |  | flagellar filament core protein |  |  | -1.75 |  | -1.77 | **-2.23** |  |
|  |  |  |  |  |  |  |  |  |  |  |
| 4 | TDE1482* |  | peptidase, M24 family protein |  |  |  | 1.75 | **2.00** | **-2.16** |  |
|  |  |  |  |  |  |  |  |  |  |  |
| 7 | TDE1516 |  | ABC transporter, ATP-binding protein, putative |  |  |  |  | **2.23** |  |  |
|  |  |  |  |  |  |  |  |  |  |  |
| 8 | TDE1548 |  | conserved hypothetical protein TIGR00103 |  |  | **9.24** |  |  |  |  |
|  |  |  |  |  |  |  |  |  |  |  |
| 6 | TDE1556 |  | conserved domain protein | **2.00** |  |  | 1.88 |  |  |  |
|  |  |  |  |  |  |  |  |  |  |  |
| 8 | TDE1593 |  | Fe-hydrogenase |  |  |  | -1.70 |  |  |  |
| 8 | TDE1594 |  | pyridine nucleotide-disulphide oxidoreductase family protein |  |  |  | **-2.16** |  |  |  |
|  |  |  |  |  |  |  |  |  |  |  |
| 3 | TDE1624* | *gcvP2* | glycine cleavage system P protein, subunit 2 | **-2.05** |  | -1.68 | **-2.23** | **-2.31** | **-2.03** |  |
| 3 | TDE1625* | *gcvP1* | glycine cleavage system P protein, subunit 1 | -1.82 |  | - | **-2.17** | **-2.32** | **-2.08** |  |
| 3 | TDE1626* | *gcvH* | glycine cleavage system H protein | -1.77 |  | - | -1.63 | **-2.23** | **-2.17** |  |
| 3 | TDE1627* | *gcvT* | glycine cleavage system T protein | - |  | -1.59 | -1.81 | **-2.30** | **-2.46** |  |
|  |  |  |  |  |  |  |  |  |  |  |
| 5 | TDE1663* |  | OmpA family protein |  | 1.95 |  |  | **-2.14** | **-2.04** |  |
| 5 | TDE1664* |  | conserved domain protein |  | + |  |  | -1.69 | -1.93 |  |
|  |  |  |  |  |  |  |  |  |  |  |
| 3 | TDE1677 | *ssb* | single-strand binding protein | -1.52 | 1.50 |  | **2.06** | **-2.27** | **-2.34** |  |
| 3 | TDE1678 | *rpsF* | ribosomal protein S6 |  |  |  | **2.10** | **-2.32** | **-2.22** |  |
|  |  |  |  |  |  |  |  |  |  |  |
| 7 | TDE1712* | *flaA* | flagellar filament outer layer protein |  |  |  |  |  | **-2.05** |  |
|  |  |  |  |  |  |  |  |  |  |  |
| 7 | TDE1717* |  | hypothetical protein |  |  |  |  | -1.93 | **-2.88** |  |
|  |  |  |  |  |  |  |  |  |  |  |
| 5 | TDE1722 |  | hypothetical protein | **3.30** |  |  |  |  | -1.50 |  |
|  |  |  |  |  |  |  |  |  |  |  |
| 5 | TDE1830 |  | hypothetical protein | 1.58 |  |  |  |  | **2.44** |  |
|  |  |  |  |  |  |  |  |  |  |  |
| 5 | TDE1838 |  | conserved hypothetical protein | **2.01** |  |  |  | -1.64 |  |  |
|  |  |  |  |  |  |  |  |  |  |  |
| 7 | TDE1947 |  | ABC transporter, permease protein |  |  |  |  | - | **-2.18** |  |
|  |  |  |  |  |  |  |  |  |  |  |
| 7 | TDE1950* |  | membrane lipoprotein TmpC, putative |  |  |  |  | **-2.43** | -1.66 |  |
|  |  |  |  |  |  |  |  |  |  |  |
| 5 | TDE1961 |  | PIN domain protein | 1.77 |  |  |  | **4.92** |  |  |
|  |  |  |  |  |  |  |  |  |  |  |
| 4 | TDE1978 |  | conserved hypothetical protein |  |  |  | **-2.12** | - | - |  |
| 4 | TDE1979 |  | hypothetical protein |  |  |  | **-2.14** | -1.53 | -1.58 |  |
| 4 | TDE1980 |  | hypthetical protein |  |  |  | -1.57 | + | - |  |
|  |  |  |  |  |  |  |  |  |  |  |
| 7 | TDE2001 |  | oligoendopeptidase F, putative |  |  |  |  |  | **-2.31** |  |
|  |  |  |  |  |  |  |  |  |  |  |
| 8 | TDE2006 |  | membrane protein, putative |  |  |  | **2.56** |  |  |  |
| 8 | TDE2007 |  | ABC transporter, ATP-binding/permease protein |  |  |  | **3.17** |  |  |  |
| 8 | TDE2008 |  | ABC transporter, ATP-binding/permease protein |  |  |  | **2.45** |  |  |  |
| 8 | TDE2009 |  | conserved hypothetical protein |  |  |  | **4.03** |  |  |  |
|  |  |  |  |  |  |  |  |  |  |  |
| 4 | TDE2054 |  | conserved hypothetical protein |  |  |  | **2.68** | -1.86 | -1.89 |  |
| 4 | TDE2055* | *hbpB* | hemin-binding protein B |  |  |  | **2.43** | **-2.11** | **-2.00** |  |
| 4 | TDE2056* |  | outer membrane hemin-binding protein A |  |  |  | **2.98** | **-2.29** | **-2.85** |  |
|  |  |  |  |  |  |  |  |  |  |  |
| 4 | TDE2078 |  | TPR domain protein |  |  |  | 1.53 | - | -1.58 |  |
| 4 | TDE2079 |  | sigma-54 dependent transcriptional regulator, putative |  |  |  | 1.68 | - | - |  |
| 4 | TDE2080 |  | cytidylate kinase/ribosomal protein S1 |  |  |  | **2.10** | -1.98 | **-2.05** |  |
|  |  |  |  |  |  |  |  |  |  |  |
| 8 | TDE2093 |  | conserved hypothetical protein |  |  |  | **-2.30** |  |  |  |
|  |  |  |  |  |  |  |  |  |  |  |
| 7 | TDE2118 |  | topoisomerase IV, A subunit, putative |  |  |  |  | **3.72** |  |  |
|  |  |  |  |  |  |  |  |  |  |  |
| 5 | TDE2119* | *grdB-2* | glycine reductase complex selenoprotein GrdB2 | -1.73 |  |  |  | **-2.32** | - |  |
| 5 | TDE2120* |  | glycine reductase complex proprotein GrdE2 | -1.65 |  |  |  | **-2.29** | -1.93 |  |
|  |  |  |  |  |  |  |  |  |  |  |
| 4 | TDE2180 | *trmE* | tRNA modification GTPase TrmE |  |  | 1.52 |  | -1.50 | **-3.04** |  |
|  |  |  |  |  |  |  |  |  |  |  |
| 4 | TDE2200* | *megL* | methionine gamma-lyase |  |  |  | **-2.78** | **-2.24** | -1.86 |  |
|  |  |  |  |  |  |  |  |  |  |  |
| 3 | TDE2214 |  | conserved hypothetical protein |  | 1.63 |  | 1.67 | -1.88 | **-2.68** |  |
|  |  |  |  |  |  |  |  |  |  |  |
| 7 | TDE2217* | *mglB* | galactose/glucose-binding lipoprotein |  |  |  |  | **-2.09** | **-2.20** |  |
|  |  |  |  |  |  |  |  |  |  |  |
| 7 | TDE2232 |  | iron compound ABC transporter, ATP-binding protein, putative | 1.64 |  | 1.88 |  |  |  |  |
| 7 | TDE2233 |  | iron compound ABC transporter, permease protein, putative |  |  |  |  |  | - |  |
| 7 | TDE2234* |  | iron compound ABC transporter, periplasmic iron  compound-binding protein, putative |  |  |  |  |  | - | **-2.03** |
| 7 | TDE2235* |  | methylaspartate ammonia-lyase |  |  |  |  | **-2.38** | **-2.20** |  |
| 7 | TDE2236* |  | methylaspartate mutase, E subunit |  |  |  |  | -1.97 | -1.82 |  |
|  |  |  |  |  |  |  |  |  |  |  |
| 7 | TDE2271 |  | HAM1 protein |  |  |  |  |  | **-2.15** |  |
|  |  |  |  |  |  |  |  |  |  |  |
| 7 | TDE2285 |  | conserved hypothetical protein |  |  |  |  | **-2.35** | **-3.14** |  |
|  |  |  |  |  |  |  |  |  |  |  |
| 3 | TDE2300* |  | trypsin domain/PDZ domain protein | 1.96 |  |  | **2.42** | -1.55 | -1.67 |  |
|  |  |  |  |  |  |  |  |  |  |  |
| 7 | TDE2315* |  | conserved hypothetical protein TIGR00044 |  |  |  |  | -1.55 | **-2.83** |  |
|  |  |  |  |  |  |  |  |  |  |  |
| 7 | TDE2326 |  | cobyric acid synthase CobQ, putative |  |  |  |  |  | **-2.12** |  |
|  |  |  |  |  |  |  |  |  |  |  |
| 6 | TDE2327 | *clpB* | ATP-dependent Clp protease, ATP-binding subunit ClpB | **2.20** |  |  | 1.53 |  |  |  |
|  |  |  |  |  |  |  |  |  |  |  |
| 5 | TDE2369* |  | conserved domain protein |  | 1.52 |  |  | **-2.11** | **-2.37** |  |
|  |  |  |  |  |  |  |  |  |  |  |
| 8 | TDE2372 |  | conserved hypothetical protein |  |  |  | **2.83** |  |  |  |
|  |  |  |  |  |  |  |  |  |  |  |
| 8 | TDE2398 |  | conserved hypothetical protein TIGR00278 |  |  |  | 1.92 |  |  |  |
| 8 | TDE2399 | *rnpA* | ribonuclease P protein component |  |  |  | **2.36** |  |  |  |
| 8 | TDE2400 |  | ribosomal protein L34 |  |  |  | 1.50 |  |  |  |
|  |  |  |  |  |  |  |  |  |  |  |
| 8 | TDE2410 |  | hemolysin |  |  |  | **-2.29** |  |  |  |
|  |  |  |  |  |  |  |  |  |  |  |
|  | TDE2423 |  | ribosomal protein L10 | -1.52 |  |  |  | -1.58 | -1.62 |  |
|  | TDE2424 | *rplA* | ribosomal protein L1 | - |  |  |  | -1.88 | **-2.12** |  |
|  | TDE2425 |  | ribosomal protein L11 | -1.69 |  |  |  | - | -1.40 |  |
|  |  |  |  |  |  |  |  |  |  |  |
| 5 | TDE2429 |  | hypothetical protein | **2.00** |  |  |  | **9.96** | 1.67 |  |
|  |  |  |  |  |  |  |  |  |  |  |
| 4 | TDE2465 |  | hypothetical protein |  |  | - | **-2.90** | -1.61 | -1.77 |  |
| 4 | TDE2466 |  | conserved hypothetical protein |  |  | -1.53 | **-3.30** | -1.58 | -1.85 |  |
| 4 | TDE2467 |  | conserved domain protein |  |  | -1.67 | **-2.59** | -1.87 | -1.91 |  |
|  |  |  |  |  |  |  |  |  |  |  |
| 5 | TDE2508* |  | hypothetical protein | -1.94 |  |  |  | -1.84 | **-2.19** |  |
|  |  |  |  |  |  |  |  |  |  |  |
| 7 | TDE2557 |  | hypothetical protein |  |  |  |  |  | **-2.19** |  |
|  |  |  |  |  |  |  |  |  |  |  |
| 4 | TDE2601* |  | surface antigen, putative |  |  |  | 1.52 | - | - |  |
| 4 | TDE2602* |  | outer membrane protein, putative |  |  |  | 1.59 | -1.71 | **-2.01** |  |
|  |  |  |  |  |  |  |  |  |  |  |
| 7 | TDE2674 |  | hypothetical protein |  |  |  |  | -1.84 | **-2.42** |  |
|  |  |  |  |  |  |  |  |  |  |  |
| 7 | TDE2712* |  | hypothetical protein |  |  |  |  |  | **-2.00** |  |
|  |  |  |  |  |  |  |  |  |  |  |
| 7 | TDE2739 |  | membrane protein, putative |  |  |  |  | - | **-2.00** |  |

See legend Table 3. * indicates that these genes were identified as putative surface antigens by Veith *et. al* 2009.
