# Supplementary material for: Transcriptional Responses of Treponema denticola to Other Oral Bacterial Species
Source: PLoS One. 2014 Feb 5;9(2):e88361. doi: 10.1371/journal.pone.0088361 (PMC3914990; doi:10.1371/journal.pone.0088361)
Supplement: Table S2 — See legend Table 3 . MA = values derived from microarray experiments, RT = values derived from real-time PCR experiments (DOCX) [file pone.0088361.s003.docx]

Supplemental Table S2: Validating Microarray data with RT-PCR.

| **Complex** |  | **Yellow** | | | **Orange** | | | | **Red** | | | |  |
| --- | --- | --- | --- | --- | --- | --- | --- | --- | --- | --- | --- | --- | --- |
|  |  | ***Sg*** | | ***Ss*** | | ***Fn*** | | ***Pi*** | | ***Pg*** | | ***Tf*** | |
| **Locus** | **Predicted Gene Product** | **MA** | **RT** | **MA** | **RT** | **MA** | **RT** | **MA** | **RT** | **MA** | **RT** | **MA** | **RT** |
|  |  |  |  |  |  |  |  |  |  |  |  |  |  |
| TDE0358 | cinnamoyl ester hydrolase | **4.04** | **2.67** |  | **2.98** |  |  |  |  |  |  | **-2.12** |  |
|  |  |  |  |  |  |  |  |  |  |  |  |  |  |
| TDE0405 | major outer sheath protein | **-2.92** | **-6.7** |  | **-5.4** | -1.52 | - | -1.66 |  |  |  | **-2.12** |  |
|  |  |  |  |  |  |  |  |  |  |  |  |  |  |
| TDE0449 | ferritin, putative |  |  |  | 1.76 |  |  | 1.52 | + |  | 1.98 | **5.62** | + |
|  |  |  |  |  |  |  |  |  |  |  |  |  |  |
| TDE1004 | flagellar filament core protein | **-2.23** | **-5.6** |  | **-3.3** |  |  | 1.58 |  | -1.54 | - | **-2.02** |  |
|  |  |  |  |  |  |  |  |  |  |  |  |  |  |
| TDE1028 | hypothetical protein |  |  |  |  |  |  | **4.96** | **2.34** |  | 1.56 |  |  |
| TDE1029 | Hsp20/alpha rystalline family protein | **2.34** |  |  |  |  |  |  | 1.56 |  |  |  |  |
|  |  |  |  |  |  |  |  |  |  |  |  |  |  |
| TDE1072 | lipoprotein, putative |  |  |  |  |  |  | **-2.72** | - | **-2.67** |  | **-2.64** |  |
|  |  |  |  |  |  |  |  |  |  |  |  |  |  |
| TDE1238 | preprotein translocase, SecG subunit |  |  |  |  |  |  |  |  | **-2.38** | -1.67 | **-3.05** | -1.73 |
|  |  |  |  |  |  |  |  |  |  |  |  |  |  |
| TDE1408 | flagellar filament outer layer protein FlaA, putative | -1.66 | **-2.74** |  | **-2.09** | -1.62 | **-5.67** |  | **-2.67** | **-2.38** | **-3.98** | **-3.45** | **-6.45** |
|  |  |  |  |  |  |  |  |  |  |  |  |  |  |
| TDE1548 | conserved hypothetical protein |  |  |  | - | **9.24** | **2.42** |  | 1.95 |  | 1.73 |  |  |
|  |  |  |  |  |  |  |  |  |  |  |  |  |  |
| TDE1722 | hypothetical protein | **3.30** |  |  |  |  |  |  |  |  |  | -1.50 |  |
|  |  |  |  |  |  |  |  |  |  |  |  |  |  |
| TDE2009 | conserved hypothetical protein |  | - |  | - |  | **2.66** | **4.03** | 1.89 |  | **2.47** |  | **2.32** |

See legend Table 3. MA = values derived from microarray experiments, RT = values derived from real-time PCR experiments
